# High diagnostic concordance between vulvar and cervical samples for human papillomavirus detection: a cross-sectional study

**DOI:** 10.1128/spectrum.04026-25

**Published:** 2026-08-14

**Authors:** Shuying Miao, Wenyan Guan, Wen Jiang, Biao Zhang, Jun Yang, Linyue Zhao, Lu He

**Affiliations:** 1 Department of Pathology, Nanjing Drum Tower Hospital, Affiliated Hospital of Medical School, Nanjing University 12581, Nanjing, China; Central Texas Veterans Health Care System, Temple, Texas, USA

**Keywords:** human papillomavirus, vulvar sampling, cervical HPV testing, non-invasive sampling, HPV genotyping, vulvar-cervical concordance, negative predictive value

## Abstract

Low compliance with clinician-collected cervical sampling and implementation barriers in resource-limited settings limit human papillomavirus (HPV)-based cervical cancer prevention. Vulvar sampling is non-invasive and easier to perform, but its agreement with cervical HPV testing remains insufficiently defined. We retrospectively analyzed 719 paired vulvar and cervical specimens tested by clinically validated fluorescence PCR to evaluate concordance between sampling sites and the potential utility of vulvar HPV testing. High-risk HPV (hrHPV) positivity was 47.98% (345/719) in vulvar samples and 43.53% (313/719) in cervical samples, with strong site-to-site concordance (κ = 0.821). The negative predictive value of vulvar hrHPV testing for concurrent cervical hrHPV positivity was 95.72%. Concordance was highest for HPV18 (κ = 0.892; negative predictive value [NPV] = 99.41%), followed by HPV16 (κ = 0.839), and low-risk HPV6/11 (κ = 0.746). Among five biopsy-confirmed cervical intraepithelial neoplasia cases, vulvar and cervical HPV results were fully concordant. Age-stratified analysis showed peak hrHPV infection in women younger than 30 years. In women aged 40–49 years, vulvar hrHPV positivity exceeded cervical positivity (56.52% vs 45.65%). Agreement was lower among women aged ≥40 years for hrHPV and lrHPV testing. Among discordant cases, 93.75% (15/16) of vulvar-negative/cervical-positive cases had prior HPV-related treatment, whereas non-HPV16/18 types predominated in vulvar-positive/cervical-negative cases (91.67%, 44/48). Vulvar HPV testing showed high concordance with paired cervical testing and may help identify women at low likelihood of concurrent cervical hrHPV positivity. These findings support prospective evaluation of vulvar sampling as a non-invasive adjunct for HPV testing, particularly for women with low acceptance of cervical sampling or limited access to conventional screening.

**IMPORTANCE:**

Conventional cervical human papillomavirus (HPV) screening remains inaccessible to some women because of discomfort, perceived invasiveness, privacy concerns, and implementation barriers. This study shows that HPV testing with vulvar swabs, a truly non-invasive sampling site, has high concordance with paired cervical testing (κ = 0.821). The high negative predictive value of vulvar high-risk HPV (hrHPV) testing for concurrent cervical hrHPV negativity (95.72%) suggests that a negative vulvar result may identify women at low likelihood of cervical hrHPV positivity, potentially reducing unnecessary referrals. These findings are relevant for older and postmenopausal women, in whom cervical atrophy may decrease the sensitivity of HPV detection. Overall, our results provide preliminary support for prospective evaluation of vulvar sampling as a patient-friendly adjunct to HPV testing, particularly for women with low acceptance of cervical sampling or limited access to conventional screening. Population-based validation with cytological and histological endpoints is required before broader implementation.

## INTRODUCTION

Human papillomavirus (HPV) infection is a prevalent female lower genital tract pathogen, and persistent infection with high-risk HPV types is the principal cause of cervical carcinogenesis (1, 2). Cervical cancer ranks fourth among female malignancies globally, accounting for approximately 7% of all cancers in women, and 84% to 90% of cases occur in low- and middle-income countries (3–6). Although HPV-based screening has substantially reduced the incidence and mortality of cervical cancer, low coverage in resource-limited regions, compounded by technical costs, leaves many women unserved (7–10).

Clinician-collected cervical specimens remain the standard sampling approach for HPV testing. However, cervical sampling requires speculum examination and direct collection from the cervix, which may cause discomfort, embarrassment, or reluctance among some women, thereby limiting screening participation. In addition, traditional cervical cytology sampling may impair epithelium, increasing HPV susceptibility (11, 12). To overcome these barriers, vaginal self-sampling for HPV testing has been increasingly introduced into cervical cancer screening programs and has been shown to effectively improve screening uptake (11–13). Nevertheless, vaginal self-sampling still requires insertion of a sampling device into the vagina, and some women may remain unwilling or unable to perform this procedure correctly. It also carries risks of mucosal injury (14–16). Compared with cervical or vaginal sampling, vulvar sampling is easier to perform, does not require speculum examination or intravaginal insertion, and may therefore represent a more acceptable and feasible non-invasive sampling approach. However, evidence regarding the concordance between vulvar HPV testing and paired cervical HPV testing remains limited, and the potential role of vulvar sampling in HPV detection has not been fully evaluated.

This study aims to evaluate the concordance between vulvar and cervical HPV detection in a large cohort, compare HPV positivity patterns between the two sampling sites, and assess whether vulvar HPV testing may provide supportive information for identifying concurrent cervical HPV status. These findings may provide preliminary evidence for further evaluation of vulvar sampling as a non-invasive adjunctive approach for HPV testing, particularly in populations with low acceptance of cervical sampling or limited access to conventional screening resources.

## MATERIALS AND METHODS

### Study population

**Fig 1:**
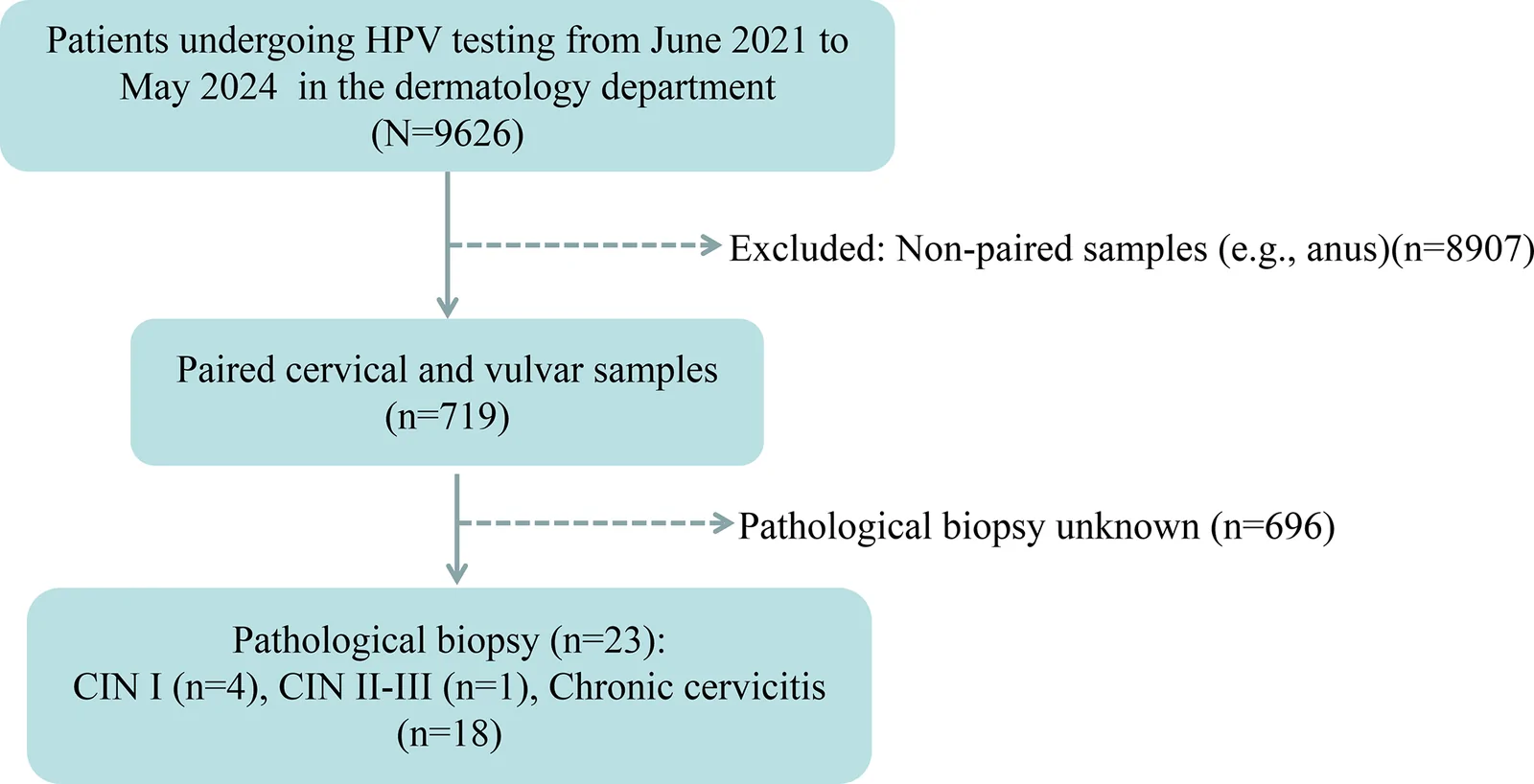
Study flowchart. HPV, human papillomavirus; CIN, cervical intraepithelial neoplasia.

During the study period from June 2021 to May 2024, a total of 9,626 female patients who visited the Department of Dermatology at Nanjing Drum Tower Hospital underwent HPV testing. Paired vulvar and cervical HPV testing was performed when dermatologists clinically suspected HPV-related anogenital lesions or infection and considered concurrent assessment of both vulvar and cervical sites necessary. Women were eligible for the present analysis if paired vulvar and cervical specimens were collected during the same clinical encounter, and both specimens underwent HPV genotyping. Based on these criteria, 719 women were included in the final study population, as shown in Fig. 1. Available clinical and laboratory variables included age, HPV genotyping results from paired vulvar and cervical samples, β-globin Ct values, histopathological findings when biopsy was performed, and history of HPV-related treatment when available. HPV vaccination history was not systematically recorded in the medical records. This retrospective analysis was based on anonymized data, and the requirement for informed consent was waived.

### Specimen collection

All samples were professionally collected by dermatologists who had received standardized training in sample collection procedures, and no patient self-collected samples were included in this study. All sampling procedures were performed using disposable sterile sampling brushes and dedicated cell preservation media that were matched to the corresponding HPV detection kits to ensure consistency of sample quality. For cervical samples, excess secretions were first removed with a cotton swab. A cervical sampling brush was then inserted into the squamocolumnar junction/transformation zone of the cervix and rotated five times for over 5 s to collect exfoliated cervical cells. The brush was subsequently placed into 1 mL of dedicated cervical cell preservation medium. For vulvar samples, a sampling brush was rotated 5 to 10 times across the vulvar area to collect exfoliated cells and was similarly preserved in dedicated medium. All procedures were carried out under aseptic conditions to minimize the risk of cross-contamination.

### DNA isolation

Samples were vortexed thoroughly to ensure homogeneous suspension. A 1 mL of the aliquot of each specimen was transferred to a 1.5 mL centrifuge tube and centrifuged at 13,000 rpm for 5 min. After removal of the supernatant, the precipitate was resuspended in 50 μL of DNA extraction buffer, mixed thoroughly, and incubated at 100°C for 10 min. The samples were then centrifuged again at 13,000 rpm for 10 min, and the supernatant was retained as the DNA template for subsequent PCR analysis.

### HPV detection and genotyping

High-risk human papillomavirus (hrHPV) detection and HPV16/18 genotyping were performed using the 14 high-risk HPV nucleic acid detection and 16/18 typing kit (Jiangsu Merkin Biotechnology Engineering Co., Ltd., China). This assay detects HPV16, HPV18, and 12 other hrHPV types, including HPV31, 33, 35, 39, 45, 51, 52, 56, 58, 59, 66, and 68. LrHPV detection was performed using the HPV6/11 nucleic acid detection kit (Wuhan Baitai Gene Engineering Co., Ltd., China).

Human β-globin amplification was included as an internal control to assess specimen adequacy and the presence of PCR inhibition. Only samples with valid β-globin amplification were considered adequate for HPV testing. Samples with invalid β-globin amplification were considered inadequate and were excluded or repeated according to the laboratory protocol. PCR amplification and fluorescence detection were performed using an ABI 7500 real-time PCR system (Applied Biosystems, USA).

For the hrHPV assay, the PCR program consisted of uracil-N-glycosylase treatment at 25°C for 10 min and initial denaturation/enzyme activation at 95°C for 10 min, followed by 40 cycles of 95°C for 15 s, 60°C for 30 s, and 40°C for 1 min. According to the manufacturer’s instructions, hrHPV positivity was defined as amplification of the corresponding HPV target with a cycle threshold (Ct) value of ≤37 in the presence of a valid β-globin internal control signal (β-globin Ct ≤37). Samples with no detectable HPV amplification or an HPV Ct value >37, together with a valid β-globin signal, were interpreted as hrHPV-negative.

For the lrHPV assay targeting HPV6/11, PCR was performed on the same ABI 7500 system under the following conditions: 50°C for 2 min and 95°C for 3 min, followed by 40 cycles of 95°C for 10 s and 60°C for 1 min, with fluorescence acquisition at the 60°C step. The reporter dye was FAM, with ROX used as the passive reference dye. HPV6/11 positivity was defined as an S-shaped amplification curve with a Ct value of ≤37.00. Samples with Ct values >37.00 or no amplification were considered negative, whereas results in the Ct range of 37.00–40.00 were retested according to the manufacturer’s protocol.

Overall, hrHPV positivity was defined as detection of any of the 14 high-risk HPV subtypes, whereas lrHPV positivity was defined as detection of HPV6 and/or HPV11.

### Histopathological assessment

Cervical biopsy results were available for 23 hrHPV-positive patients and were abstracted from the medical records. Among these patients, four were diagnosed with CIN grade I, one with CIN grades II–III, and 18 with chronic cervicitis.

### Statistical analysis

Statistical analyses were performed using GraphPad Prism 7.0 and SPSS 26.0. Categorical variables were summarized as frequencies and percentages (%). Comparisons between independent groups were performed using the *χ*² test or Fisher’s exact test, as appropriate. Continuous variables were expressed as mean ± standard deviation or median with interquartile range, depending on data distribution, and were compared using Student’s *t*-test or the Mann-Whitney *U* test, as appropriate. Cervical HPV testing was used as the reference comparator for calculating the sensitivity, specificity, positive predictive value (PPV), and negative predictive value (NPV) of vulvar HPV testing. Agreement between cervical and vulvar HPV testing was assessed using Cohen’s kappa coefficient, which accounts for agreement occurring by chance. All statistical tests were two-sided, and significance was defined as *P* < 0.05.

## RESULTS

### Participant characteristics and overall HPV positivity

A total of 719 women were included in the final analysis. Each participant provided paired vulvar and cervical specimens, yielding 1,438 specimens (Fig. 1), all of which met the PCR validity criteria based on successful β-globin amplification. The age of the participants ranged from 14 to 71 years. Overall, hrHPV positivity peaked in women aged <30 years. The distribution of HPV positivity categories was broadly similar between vulvar and cervical specimens. Across both sampling sites, the most frequently detected categories were lrHPV positivity, hrHPV positivity, hrHPV/lrHPV co-positivity, HPV16 positivity, and HPV16/18 co-positivity, in descending order of frequency (Table 1).

**TABLE 1:** Participant characteristics and HPV positivity rates

Characteristic	All (*N* = 719)	Vulvar hrHPV + *n* (%)	Cervical hrHPV + *n* (%)	*P* value
Age (years)^*a*^				
<30	430 (59.81)	211 (61.16)	200 (63.90)	0.495
30–39	198 (27.54)	88 (25.51)	75 (23.96)	0.220
40–49	46 (6.40)	26 (7.54)	21 (6.71)	0.404
≥50	45 (6.26)	20 (5.80)	17 (5.43)	0.669
Total hrHPV+		345 (47.98)	313 (43.53)	0.101
hrHPV subtypes				
OHR^*b*^		253 (35.19)	224 (31.15)	0.117
HPV 16		53 (7.37)	50 (6.95)	0.838
HPV 18		32 (4.45)	34 (4.73)	0.900
HPV 16/18		7 (0.97)	5 (0.70)	0.774
lrHPV+		353 (49.10)	314 (43.67)	**0.046^*c*^**
hr/lr co-infection+		191 (26.56)	170 (23.64)	0.223

^*a*^ Age distribution among hrHPV-positive participants.

^*b*^ Other hrHPV (non-HPV16/18); infection/prevalence peak refers to the group with the highest infection rate.

^*c*^ Bold indicates *P* < 0.05.

### Age-related HPV positivity patterns

**Fig 2:**
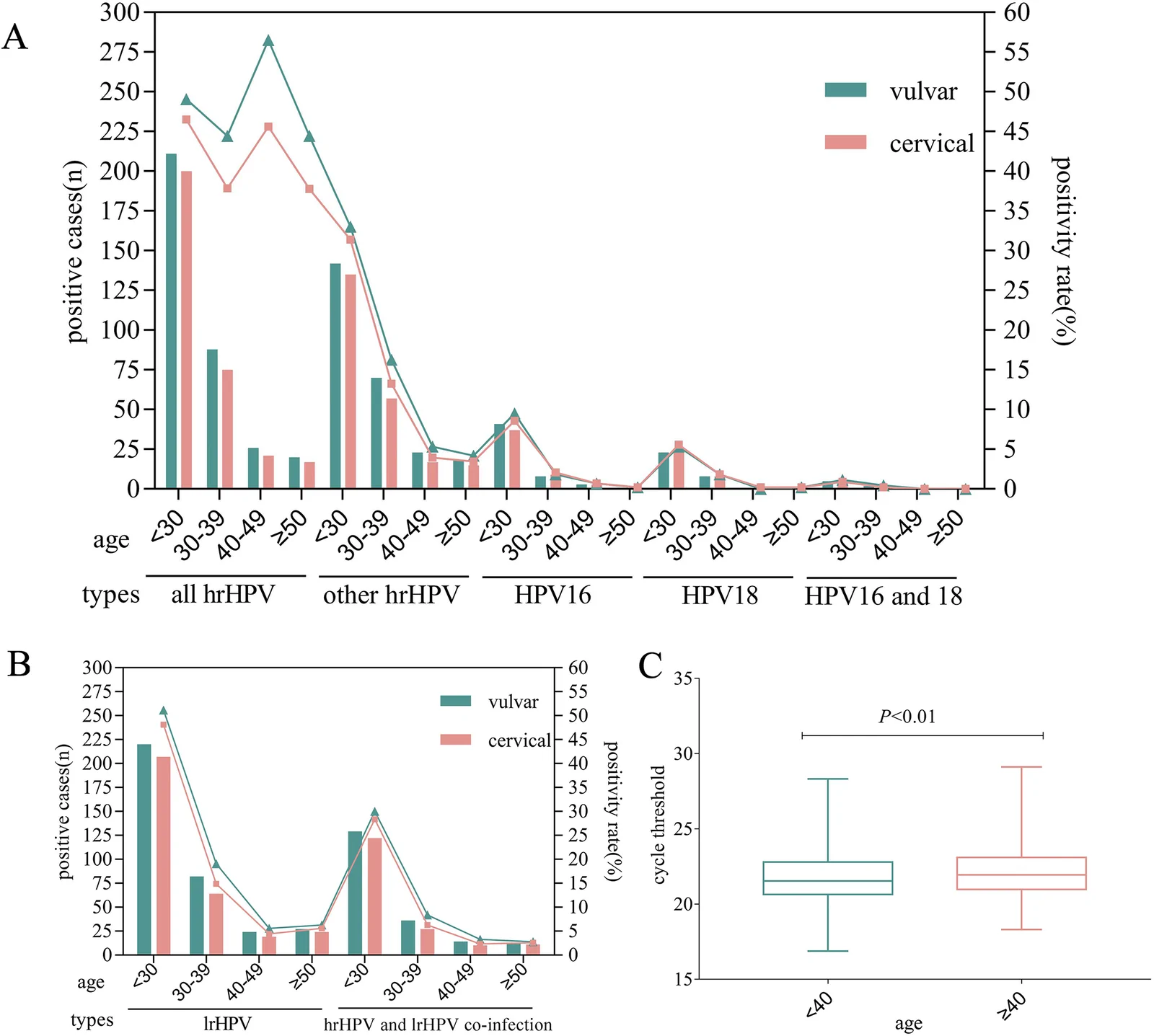
Distribution of HPV infection in vulvar and cervical samples by age group. (**A**) hrHPV positivity rate. (**B**) lrHPV positivity rate and hrHPV/lrHPV co-infection rate. (**C**) Comparison of β-globin Ct values in cervical samples between women aged <40 and ≥40 years (Mean ± SEM, <40: 21.81 ± 0.056; ≥40: 22.14 ± 0.0567; *P* < 0.01 by *t*-test).

Age-stratified analysis showed that HPV16 and HPV18 positivity tended to decline in women aged ≥40 years. Across all age groups, the vulvar hrHPV positivity rate was higher than the cervical hrHPV positivity rate. The largest difference was observed in women aged 40–49 years, in whom the vulvar hrHPV positivity rate was 56.52%, compared with 45.65% for cervical specimens, corresponding to an absolute difference of 10.87% (Fig. 2A and B). We also compared β-globin Ct values in cervical specimens between age groups to evaluate age-related differences in internal-control amplification. Cervical β-globin Ct values were slightly but significantly lower in women aged <40 years than in those aged ≥40 years (21.81 ± 0.056 vs 22.14 ± 0.0567, *P* < 0.01; Fig. 2C), confirming better cellularity in younger patients.

### Concordance between vulvar and cervical HPV testing

Among the 719 pairs, 655 showed concordant hrHPV results, corresponding to an overall concordance rate of 91.10%. Discordant hrHPV results were observed in 64 pairs (8.90%). Using cervical HPV testing as the reference comparator, vulvar hrHPV testing showed a sensitivity of 94.89%, specificity of 88.18%, PPV of 86.09%, and NPV of 95.72%. Agreement between vulvar and cervical hrHPV testing was strong, with a Cohen’s kappa coefficient (κ) of 0.821. The high NPV indicates that a negative vulvar hrHPV result was strongly associated with concurrent cervical hrHPV negativity in this selected clinical cohort.

Subtype-specific analysis showed the highest concordance for HPV18, with a kappa coefficient of 0.892. For HPV18 detection, vulvar testing showed a sensitivity of 89.74%, specificity of 99.41%, PPV of 89.74%, and NPV of 99.41%. High concordance was also observed for HPV16 detection (κ = 0.839) and lrHPV detection (κ = 0.746) (Table 2).

**TABLE 2:** Concordance between vulvar and cervical samples for HPV detection^*a*^

HPV type/group	Concordance pattern (vulvar/cervical)				Sensitivity (%)	Specificity (%)	PPV (%)	NPV (%)	κ	*P* value
	+/+	+/−	−/+	−/−						
hrHPV										
All	297	48	16	358	94.89	88.18	86.09	95.72	0.821	**<0.01^*b*^**
HPV 16	49	11	6	653	89.09	98.34	81.67	99.09	0.839	**<0.01**
HPV 18	35	4	4	676	89.74	99.41	89.74	99.41	0.892	**<0.01**
lrHPV	288	65	26	340	91.72	83.95	81.59	92.90	0.746	**<0.01**
Age stratification (hrHPV)										
<40 years	260	39	15	314	94.54	88.95	86.96	95.44	0.827	**<0.01**
≥40 years	37	9	1	44	97.37	83.02	80.43	97.78	0.781	**<0.01**
Age stratification (lrHPV)										
<40 years	265	84	36	243	88.03	74.31	75.93	87.10	0.620	**<0.01**
≥40 years	43	32	10	6	81.13	15.79	57.33	37.50	−0.034	0.704

^*a*^ +/+: both vulva and cervix positive; +/−: vulva positive, cervix negative; −/+: vulva negative, cervix positive; −/−: both negative.

^*b*^ Bold indicates *P* < 0.05.

Among the 23 hrHPV-positive patients who underwent cervical biopsy, 5 were diagnosed with CIN, including 4 cases of CIN I and 1 case of CIN II–III. All five biopsy-confirmed CIN cases showed concordant vulvar and cervical HPV results, corresponding to a concordance rate of 100%. Given the small number of biopsy-confirmed CIN cases, this finding was interpreted descriptively.

Age-stratified concordance analysis showed that agreement between vulvar and cervical testing varied by age (Table 2). For hrHPV, the kappa coefficient was lower in women aged ≥40 years than in those aged <40 years (κ = 0.781 vs 0.827). For lrHPV, concordance was substantially lower in women aged ≥40 years than in those aged <40 years (κ = −0.034 vs 0.620), suggesting that agreement for lrHPV detection may be more sensitive to age distribution, sample size, or site-specific detection patterns.

### HPV16/18 distribution in discordant samples

We next examined HPV16/18 distribution among discordant sample pairs. Vulvar-positive/cervical-negative (V+/C−) cases showed significantly lower HPV16/18 positivity than the other hrHPV-positive groups (8.33% vs 25.00–29.07%, all *P* < 0.05), indicating that V+/C− discordance was predominantly driven by non-HPV16/18 types (91.67%). In vulvar-negative/cervical-positive (V−/C+) cases, HPV16/18 positivity was 25.00% (Table 3). This observation indicates that a subset of cervical HPV-positive cases, including HPV16/18-positive infections, may not be captured by vulvar sampling. Therefore, vulvar-negative results should be interpreted cautiously, particularly when clinical suspicion remains high or when cervical disease evaluation is indicated.

**TABLE 3:** HPV16/18 positivity in vulvar and cervical hrHPV-positive samples

Sample group^*a*^	Total hrHPV+	16/18^*b*^ *n* (%)	Non-16/18 *n* (%)	*P* value^*c*^
C+	313	91 (29.07)	222 (70.93)	**0.001^*d*^**
V+	345	92 (26.67)	253 (73.33)	**0.004**
V+/C+	353	98 (27.76)	255 (72.24)	**0.003**
V−/C+	16	4 (25.00)	12 (75.00)	0.099
V+/C−	48	4 (8.33)	44 (91.67)	

^*a*^ C: cervical samples; V: vulvar samples.

^*b*^ Positive for HPV 16 or 18 or both.

^*c*^ Fisher's exact test compared to the V+/C− group.

^*d*^ Bold indicates *P* < 0.05.

### Clinical characteristics of discordant samples

We further compared clinical characteristics between V+/C− and V−/C + discordant cases. Age did not differ significantly between the two groups (32.25 ± 1.34 vs 28.75 ± 1.43, *P* = 0.161). However, the distribution of discordant patterns varied by age group. Among women aged ≥40 years with discordant results, V+/C− cases accounted for 90.00%, whereas V−/C + cases accounted for 10.00%. In contrast, among women aged <40 years with discordant results, V+/C− and V−/C + cases accounted for 72.22% and 27.78%, respectively. Although this difference was not statistically significant, it suggests that age may modestly influence the pattern of discordance between vulvar and cervical HPV testing.

A history of HPV-related treatment was significantly more frequent in V−/C + cases than in vulvar-positive/cervical-negative cases (93.75% vs 54.17%, *P* = 0.006; Table 4). This finding suggests that previous HPV-related treatment may be associated with discordant vulvar and cervical HPV results, possibly by altering local viral burden, site-specific HPV detectability, or the anatomical distribution of detectable HPV DNA. The recorded treatments included cryotherapy, topical medication, and electrocautery, as summarized in Table S1.

**TABLE 4:** Clinical characteristics of discordant vulvar-cervical HPV sample pairs

Characteristic	Vulva vs cervix		*P* value^*c*^
	+/− (*n* = 48)	−/+ (*n* = 16)	
Age (years), Mean ± SEM	32.25 ± 1.34	28.75 ± 1.43	0.161^*a*^
Age groups			
<40 years	39 (72.22%)	15 (27.78%)	0.429^*b*^
≥40 years	9 (90.00%)	1 (10.00%)	
History of HPV-positive treatment			
Yes	26 (54.17%)	15 (93.75%)	**0.006** ^*b*^
No	22 (45.83%)	1 (6.25%)	

^*a*^ Student's *t*-test.

^*b*^ Fisher's exact test.

^*c*^ Bold indicates *P* < 0.05.

To evaluate whether specimen adequacy influenced concordance, we compared β-globin Ct values between concordant and discordant cervical samples. Cervical-discordant cases had slightly higher internal control Ct values than cervical-concordant cases (21.13 ± 0.24 vs 20.49 ± 0.13, *P* = 0.009, Fig. S1). However, the effect size was small (Cohen’s d = −0.27), indicating only a modest difference in specimen adequacy between the two groups.

## DISCUSSION

To our knowledge, this is one of the largest paired-sample studies in China evaluating the concordance between vulvar and cervical HPV testing. In this dermatology-based clinical cohort, vulvar HPV testing showed high concordance with paired cervical HPV testing, with strong agreement for hrHPV detection. Using cervical HPV testing as the reference comparator, vulvar hrHPV testing showed high sensitivity, specificity, and NPV. The sensitivity of vulvar hrHPV testing reached 94.89%, which is comparable to the performance reported for vaginal self-sampling in previous studies (17, 18). Subtype-specific analysis also showed strong concordance for HPV16 and HPV18, with high NPVs for hrHPV, HPV16, HPV18, and lrHPV detection.

These findings suggest that vulvar sampling may have potential as a non-invasive adjunctive approach for HPV assessment in selected clinical settings. In particular, a negative vulvar hrHPV result may help identify women with a low likelihood of concurrent cervical hrHPV positivity in this clinically enriched cohort. However, this should not be interpreted as definitive evidence for ruling out true cervical HPV infection, CIN, or cervical cancer. The five biopsy-confirmed CIN cases in this study all showed concordant vulvar and cervical HPV results, which is consistent with the overall high concordance between the two sampling sites and broadly aligns with prior studies showing comparable performance between non-cervical HPV self-sampling and clinician-collected cervical sampling for CIN2+/CIN3+ detection (19, 20). Nevertheless, only one CIN II–III case was included; therefore, our study cannot establish the diagnostic accuracy of vulvar sampling for CIN2+ detection.

The slightly higher hrHPV positivity rate in vulvar specimens than in cervical specimens may reflect differences in anatomical sampling site, local viral distribution, epithelial exfoliation, or the broader surface area covered by vulvar sampling. Previous studies have shown that HPV can be detected at multiple anogenital sites and that detection patterns may vary across vulvar, vaginal, and cervical microenvironments (21, 22). Therefore, vulvar-positive/cervical-negative results, particularly those involving non-HPV16/18 types, may reflect site-specific HPV distribution, transient detection, or lower genital tract HPV carriage rather than definite cervical infection. Vulvar HPV positivity should therefore be interpreted together with cervical findings and clinical context.

Age-stratified analysis showed that hrHPV positivity peaked in women younger than 30 years, consistent with known HPV epidemiology and exposure patterns (23–25). Vulvar hrHPV positivity exceeded cervical positivity across all age groups, with the largest difference observed among women aged 40–49 years. Cervical β-globin Ct values were slightly higher in women aged ≥40 years than in younger women, indicating a relative difference in internal-control amplification. Importantly, all included cervical specimens met PCR internal-control criteria, so this finding should not be interpreted as evidence of inadequate cervical specimen quality. Prior screening data have also reported age-related increases in unsatisfactory cervical cytology sampling (0.4% at 20 years old vs 6.8% over 60 years old) and age-related variation in relative HPV detection between non-cervical and cervical samples (19, 23). Together, these findings support further evaluation of vulvar sampling in women aged ≥40 years, particularly postmenopausal women or those who have difficulty tolerating cervical sampling.

To assess whether specimen adequacy influenced concordance, we compared cervical β-globin Ct values between concordant and discordant pairs. Although cervical-discordant cases exhibited significantly higher internal control Ct values than cervical-concordant cases, the magnitude of this difference was small (Cohen’s d = −0.27). This suggests that reduced specimen adequacy may partly contribute to discordant cervical results, but it is unlikely to fully account for the observed discordance. Discordant sample analysis further showed that V−/C + discordance was more frequent in patients with prior HPV-related treatment, whereas V+/C− discordance was predominantly associated with non-HPV16/18 types. Different HPV genotypes may differ in tissue tropism, persistence, viral burden, and oncogenic potential (26). Thus, the predominance of non-HPV16/18 types in V+/C− cases may reflect genotype-specific differences in persistence, viral burden, or site-specific detectability, whereas prior treatment may contribute to V−/C+ discordance by altering local viral burden or the distribution of detectable HPV DNA. Accordingly, vulvar-negative results should be interpreted cautiously when cervical disease evaluation is clinically indicated, particularly in patients with prior HPV-related treatment or persistent clinical suspicion.

The main practical advantage of vulvar sampling is that it does not require speculum examination, direct cervical brushing, or intravaginal insertion. This feature is consistent with broader efforts to improve screening participation through less invasive HPV testing approaches, as reflected in recent guidelines and public health discussions on HPV self-sampling (27). However, the present study evaluated clinician-collected vulvar specimens rather than self-collected samples. Therefore, the feasibility, acceptability, reproducibility, and cost-effectiveness of vulvar self-sampling require prospective evaluation before incorporation into screening algorithms can be considered.

Several limitations should be acknowledged. First, this was a single-center retrospective study conducted in a selected dermatology-based population, and the findings may not be directly generalizable to average-risk screening populations. The relatively high cervical hrHPV positivity rate in this cohort may influence PPV and NPV. Second, detailed clinical indications for HPV testing and HPV vaccination history were not systematically available. Third, histological endpoints were available for only a limited subset of patients, so this study was not powered to evaluate the diagnostic performance of vulvar sampling for CIN2+ detection. Finally, vulvar self-sampling was not directly assessed.

In summary, this study showed high vulvar-cervical HPV testing concordance and provided preliminary evidence that vulvar sampling may serve as a non-invasive adjunctive approach for HPV evaluation, particularly among women aged ≥40 years, women with low acceptance of cervical sampling, or populations with limited access to conventional screening services. Further prospective validation in population-based screening cohorts with established cervical sampling methods and clinically meaningful histological endpoints as comparators is warranted.

## Data Availability

The data sets used and/or analyzed during the current study are available from the corresponding author on reasonable request.

## SUPPLEMENTAL MATERIAL

Supplemental material spectrum.04026-25-s0001.doc Table S1 and Fig. S1.

ASM does not own the copyrights to Supplemental Material that may be linked to, or accessed through, an article. The authors have granted ASM a non-exclusive, world-wide license to publish the Supplemental Material files. Please contact the corresponding author directly for reuse.
