# Supplementary material for: High diagnostic concordance between vulvar and cervical samples for human papillomavirus detection: a cross-sectional study
Source: Microbiol Spectr. 2026 Aug 14;14(9):e04026-25. doi: 10.1128/spectrum.04026-25 (PMC13532253; doi:10.1128/spectrum.04026-25)
Supplement: Supplementary file 1 [file spectrum.04026-25-s0001.doc]

**Supplementary Table S1. History of Prior HPV-Related Treatment Among V−/C+ Discordant Cases**

| Patient No. | Age (years) | Prior HPV-related treatment type(s) |
| --- | --- | --- |
| 1 | 26 | topical medication |
| 2 | 28 | topical medication |
| 3 | 31 | topical medication |
| 4 | 33 | topical medication |
| 5 | 21 | topical medication |
| 6 | 22 | topical medication |
| 7 | 26 | topical medication |
| 8 | 30 | electrocautery |
| 9 | 31 | None |
| 10 | 26 | topical medication、cryotherapy |
| 11 | 41 | topical medication |
| 12 | 28 | topical medication |
| 13 | 37 | topical medication |
| 14 | 34 | topical medication |
| 15 | 20 | topical medication |
| 16 | 26 | topical medication |

**Supplementary Figure S1. Cervical β-globin Ct values in concordant and discordant pairs.** β-globin Ct values were analyzed in cervical samples stratified by vulvar–cervical hrHPV concordance status. *P* value was calculated by a two-tailed Mann-Whitney U test.
